# Cooking behaviours after Diabetes Prevention Program (DPP) participation among DPP participants in Baltimore, MD

**DOI:** 10.1017/S1368980023001106

**Published:** 2023-11

**Authors:** Lauren E Russell, Jillian Tse, Janice Bowie, Caroline R Richardson, Amy Trubek, Nisa Maruthur, Julia A Wolfson

**Affiliations:** 1 Johns Hopkins Bloomberg School of Public Health, Department of Health Policy and Management, Baltimore, MD, USA; 2 Johns Hopkins Bloomberg School of Public Health, Department of International Health, Baltimore, MD 21205, USA; 3 Johns Hopkins Bloomberg School of Public Health, Department of Health Behavior and Society, Baltimore, MD, USA; 4 Brown University Medical School, Department of Family Medicine, Providence, RI, USA; 5 University of Vermont, Department of Nutrition and Food Sciences, Burlington, VT, USA; 6 Johns Hopkins University School of Medicine, Division of General Internal Medicine, Baltimore, MD, USA; 7 Johns Hopkins Bloomberg School of Public Health, Department of Epidemiology, Baltimore, MD, USA; 8 Welch Center for Prevention, Epidemiology and Clinical Research, Johns Hopkins University, Baltimore, MD, USA; 9 University of Michigan School of Public Health, Department of Health Management and Policy, Ann Arbor, MI, USA

**Keywords:** Diabetes Prevention Program, Cooking behaviour, Food and cooking skills, In-depth interviews, Behaviour change

## Abstract

**Objective:**

The Diabetes Prevention Program (DPP) is a widely implemented 12-month behavioural weight loss programme for individuals with prediabetes. The DPP covers nutrition but does not explicitly incorporate cooking skills education. The objective of the current study is to describe food and cooking skills (FACS) and strategies of recent DPP participants.

**Design:**

Photo-elicitation in-depth interviews were conducted from June to August, 2021.

**Setting:**

Baltimore, MD, USA.

**Participants:**

Thirteen Black women who participated in DPP.

**Results:**

The DPP curriculum influenced participants’ healthy cooking practices. Many participants reported shifting from frying foods to air-frying and baking foods to promote healthier cooking and more efficient meal preparation. Participants also reported that their participation in DPP made them more mindful of consuming fruits and vegetables and avoiding foods high in carbohydrates, fats, sugars and Na. With respect to food skills, participants reported that they were more attentive to reading labels and packaging on foods and assessing the quality of ingredients when grocery shopping.

**Conclusions:**

Overall, participants reported changing their food preferences, shopping practices and cooking strategies to promote healthier eating after completing the DPP. Incorporating hands-on cooking skills and practices into the DPP curriculum may support sustained behaviour change to manage prediabetes and prevent development of type 2 diabetes among participants.

Prediabetes and diabetes affect 96 million and 37 million Americans, respectively,^(1)^ and disproportionately impacts Black, Hispanic and low-income Americans^(2)^. Fortunately, type 2 diabetes is preventable, and the National Diabetes Prevention Program (DPP) is an evidence-based 12-month lifestyle change programme that can reduce the risk of diabetes^(3,4)^. The DPP focuses on weight loss, healthy nutrition with caloric restriction and increased physical activity^(1,5)^. However, the DPP curriculum incorporates little discussion of food and cooking skills (FACS)^(6,7)^ and does not include any hands-on cooking skills education^(8)^. Thus, changes to cooking practices during the DPP may be more limited than other weight loss strategies such as portion control or counting calories.

To our knowledge, no studies have examined FACS and strategies among DPP participants prior to or after DPP participation. FACS are important for shaping food choices and diet quality^(6,9,10)^, including use of scratch *v*. pre-prepared or processed foods, and higher cooking frequency is associated with improved diet quality and increased consumption of fruits and vegetables^(11,12)^. Some studies of community cooking initiatives have demonstrated that cooking skills interventions can be effective in facilitating behaviour changes that can prevent diabetes^(13–16)^.Yet, the extent to which the DPP helps participants develop healthy cooking skills is less established.

The objective of this study was to describe FACS and strategies among recent DPP participants and the ways the DPP influenced their current food behaviours. The primary goal was to understand what FACS and strategies the DPP currently promotes and assess potential gaps in knowledge and behaviours that could inform a future FACS intervention to supplement the DPP.

## Methods

This qualitative study used photo-elicitation interviews to explore FACS and strategies among former DPP participants in Baltimore City, MD^(17,18)^. The study team partnered with the [Blinded for Review], which provides the DPP in collaboration with community-based organisations, to identify recent DPP participants eligible for this study. The study team utilised the consolidated criteria for reporting qualitative research (COREQ) checklist^(19)^ to guide reporting. The [Blinded for Review] Institutional Review Board approved this study.

### Recruitment and selection of participants

The team recruited participants from April to July, 2021 using a database of recent (last several years) Baltimore City DPP participants maintained by the [Blinded for Review]. First, letters describing the study were sent to recent DPP participants (*n* 93); individuals had 3 weeks to opt out of further contact (*n* 14 opted out). Next, potential participants were contacted by phone and email to provide more detail and enroll and consent interested individuals. Oral consent was obtained over the phone and at the start of interviews. The goal of the study was described to participants as an opportunity ‘to learn about cooking practices among DPP participants’ and ‘to help us develop a cooking class as part of the DPP.’

Inclusion criteria required that individuals were past participants in a DPP administered by the [Blinded for Review] in Baltimore City, ≥ 18 years old, and willing and able to take photographs of their kitchen and food with a mobile device and send them to the study team. Exclusion criteria included having already been diagnosed with type 1 or type 2 diabetes, and simultaneous participation in another study. All participants who expressed a desire to participate met the eligibility criteria. Sixteen individuals enrolled in the study, three withdrew due to health issues or insufficient time for data collection, resulting in a total sample of 13 individuals with complete data.

### Data collection

Due to the COVID-19 pandemic, all data collection was conducted virtually. First, participants submitted photos of their kitchens, inside of their refrigerators and cupboards, and several typical meals they prepared over the course of a week. Then, in-depth interviews over Zoom and phone were conducted using the photographs to guide discussion. The interview guides were influenced by existing literature^(9,18,20)^ and covered food shopping practices, food preparation techniques and barriers and facilitators to cooking healthy meals. All interviews (11 via Zoom and 2 via phone) were conducted by JAW, a white woman, and an experienced qualitative researcher; JT, an Asian woman Master’s student with qualitative training, also participated in the interviews as a notetaker. At the conclusion of the study, participants received a $60 Amazon e-gift card as compensation.

### Analysis

Interview audio recordings were professionally transcribed verbatim. One research team member (LER, a white woman Master’s student studying qualitative methods) independently analysed interview transcripts using a grounded theory approach. Coding (by hand) used an inductive and iterative approach^(21)^. Preliminary codes were created through line-by-line coding of each transcript; transcripts were revisited as new codes arose. The codes were then categorised into broad themes and key insights. Code memos were written to correspond with each transcript during the data analysis process. Code memos, themes and key insights were discussed with JAW throughout the analysis process. LER also used reflective memos during analysis to track similarities and differences between participants, and her own assumptions, positionality and biases that may influence interpretation^(22)^.

## Results

### Sample characteristics

The sample included thirteen Black women who formerly participated in the DPP in Baltimore City (Table 1). The mean age was 61 years. A majority (61·5 %) of the participants were college graduates and more than half were married or partnered. Approximately half (54 %) were employed. Nearly 62 % of participants reported an annual household income of ≥ $60 000.

**Table 1:** Socio-demographic characteristics of study participants

	Overall	
Characteristics	*n*	%
Gender		
Female	13	100
Age		
Mean	61·4	
sd	8·7	
Race and Ethnicity		
Black	13	100
Highest level of education		
Some high school	0	0
High school	1	7·7
Some college	4	30·8
College graduate	8	61·5
Marital status		
Single	5	38·5
Married/Partnered	7	53·8
Divorced/Separated/Widowed	1	7·7
Employment Status		
Full-time	6	46·2
Part-time	1	7·7
Retired/Unemployed	6	46·2
Annual household income*		
< $35 000	1	7·7
$35 000–$59 000	4	30·8
$60 000–$85 000	5	38·5
> $85 000	3	23·1

* Median household income between 2017 and 2021 in 2021 dollars was $54 124 in Baltimore City, Maryland and $81 845 in Baltimore County, Maryland^(32)^.

DPP participants discussed numerous benefits of the DPP including building community with fellow participants and feeling more empowered and in control of one’s own health decisions and outcomes. Though some members of the sample attended cooking classes as part of their DPP experience, most had not. However, a key insight from participants focussed on the use of new cooking strategies and methods to promote more efficient and healthier eating practices after the DPP. Participants cited the DPP as the reason for focussing more on consuming fruits and vegetables and avoiding foods high in carbohydrates, fats, sugars and Na. Participants also reported feeling that they cook differently than many members of their community, including family, often citing new meal preparation and cooking techniques learned during the DPP. Participants said they would have found additional cooking instruction useful during the DPP.

Participants reported specific strategies for cooking more healthfully while still making flavourful meals and being mindful of time and cost constraints. Table 2 presents exemplar quotations chosen to illustrate key findings across participant characteristics and common strategies that individuals used after the DPP. Salads were a commonly consumed meal or meal component and ways to modify salad dressings came up in multiple interviews. The DPP raised awareness of calorie and fat content of salad dressings and participants used different strategies to address that including supplementing a portion of packaged salad dressing with citrus juice or vinegar. Participants also credited the DPP with helping them enhance flavours while reducing Na consumption, often by using herbs, seasonings like garlic powder or acids such as lemon juice. Using appliances such as air fryers and Instant Pots was another common cooking strategy. Participants owned numerous kitchen tools and appliances and found them useful for saving time and reducing the amount of fat in their diets while still being able to prepare familiar foods. Additionally, participants emphasised avoiding canned fruits and vegetables and focusing on frozen and fresh products to avoid Na and other preservatives used in canned items. Participants who had cooking classes as part of their DPP noted preparing all ingredients prior to beginning to cook as a new strategy to facilitate more timely and less stressful meal preparation. DPP participants also shared that they were more attentive to reading labels and packaging and considering the quality of ingredients during their shopping process than they were before the DPP, the latter of which resulted in many participants shopping at multiple grocery stores for routine items. Finally, the DPP encouraged participants to shop around the ‘perimeter’ of the store, where stores tend to stock produce, fish, meats and dairy.

**Table 2:** Key insights describing participants cooking practices after DPP

Supplementing salad dressings *‘Yeah, a lot of times I make dressing, or I’ll use the balsamic, or I’ll take it—the Caesar. But I cut it with rice wine vinegar, or I cut it—oh, I’m bringing stuff—or I’ll cut it with the apple cider vinegar.’ (49-year-old with some college education; did not participate in DPP cooking class)* *‘I’m am a little caloric conscious. I use a teaspoonful of extra virgin olive oil, apple cider vinegar, and lemon juice.’ (65-year-old with a college degree; participated in DPP cooking class)*
Enhancing flavor without adding salt *‘They showed us in the DPP cooking where you can use different herbs to bring out the flavors in food, where you don’t have to saturate the food with a lot of salt, or cook it in a lot of oil just to make it taste good…how garlic just brings out the flavor in some foods. They showed us about fresh ginger that I wasn’t using.’ (62-year-old with a high school degree; participated in DPP cooking class)* *‘I try to stay away from salt. I may use a little season salt at times, I like to use lemon juice also as a seasoning, to help with salt and taste, but that’s pretty much it. A lot of garlic powder, a lot of onion powder, sometimes parsley…’ (68-year-old with a college degree; did not participate in DPP cooking class)*
Using healthier cooking techniques and utilizing appliances, particularly air-fryers **‘** *But I bake my food a lot, now. Like I don’t-- how they-- my family would fry a lot of food, and like fry their chicken and fry-- I bake my food a lot. And I’m like air-fryer crazy. So if I’m going to fry food, fry chicken, I just throw my pork chops or my chicken or anything, I throw it into the air-fryer. I don’t necessarily want to fry. (49-year-old with some college education; did not participate in DPP cooking class)* **‘** *DPP has me looking at things more closely now to see what I should be using as opposed to what I was using. Say for instance, I was frying a lot, now I don’t. Or something like that. Yeah, I don’t fry a lot of foods anymore. The air fryer saved me from that, ‘cause it comes out just like it’s fried. I would put my… Instead of frying my chicken, now, I would stick it in the oven or I’ll put it on the grill. So, DPP did help me with that.’ (62-year-old woman with some college education; did not participate in DPP cooking class)*
Reducing stress and time *‘It helps to put the ingredients out. It makes cooking a lot less stressful, if you have all the ingredients right there, and also you don’t have to say, ‘Oh, I don’t have this, and I gotta run to the store and get some eggs or some milk.’ (62-year-old with a high school degree; participated in DPP cooking class)* *‘Really, just… I think that the cooking class made you think about having everything right there instead of… Because I’m a person, I’ll go to the refrigerator a million times. I’ll go to the cabinet and I’ll pull out, instead of having everything for your… Everything out that you need and really have it on hand. ‘Cause it was moving, they were moving fast. And it’s a great way to do it. Have everything that you need so you’re not searching for everything. You’re not looking to think that you need everything, and the cooking class made me think about everything down to the letter when it came to your ingredients.’ (54-year-old with a college degree; participated in DPP cooking class)*
Focusing on fresh and frozen fruits and vegetables **‘** *Vegetables, I like keeping my vegetables fresh. But if I don’t have it, I will have some cans up there, and I’ll open it…But if you don’t have it and you use canned, it’s okay, but just make sure you rinse everything off really good and then you start cooking it. But don’t just pour it in a pot in that water that’s in the can. Just make sure you take something and you rinse all of that off real good. And then you start cooking it.’…They [DPP] stressed to us how it was better if you can’t get fresh, do the frozen instead of the can.’ (67-year-old with some college education; did not participate in DPP cooking class)*
Being mindful about cost and quality **‘** *Food shopping is an adventure because I’m able now to look where I wasn’t before. I look at circulars. I pay attention to where, what is in my list of ingredients that I wanna get at a particular price. I used to just go buy. Now, I shop. So I go to maybe Harris Teeter. I might go to Safeway. I might go to Aldi’s. It just depends on the ingredients that I want at the cost that I want.’ (65-year-old with a college degree; participated in DPP cooking class)*
Sticking to the perimeter **‘** *And one of the things I learned in DPP was to buy from around the… From around the edges of the store, the perimeter of the store, and that’s what I try to do. I stay out of those aisles with the canned goods and things like that.’ (62-year-old with some college education; did not participate in DPP cooking class)*

## Discussion

In this qualitative photo-elicitation interview study to understand FACS and strategies among former DPP participants, we learned that participants employed numerous cooking techniques to achieve their diet and weight loss goals. After the DPP, participants were more mindful of their food choices and described increased awareness of fruit and vegetable intake, and heightened concern about limiting consumption of fats, carbohydrates, sugars and Na. Participants also described how the DPP made them more aware of strategies for air-frying and baking foods rather than frying, which was the technique many participants learned from family cooking practices. Even without an explicit focus on cooking in the DPP curriculum, findings reveal that participants modified their cooking practices during and after the DPP.

Our findings demonstrate that participants made changes to food preparation and procurement techniques based on ‘food literacy’^(23)^ discussions highlighting meal planning, food shopping and nutrition techniques^(24,25)^ already included in the existing DPP curriculum. In our results, those who already participated in a cooking class discussed using different strategies related to organising all their ingredients at the start of their meal preparation, which helped them save time and cook more efficiently. Given participants’ enthusiasm for more cooking-related content, which has also been noted in other studies^(26,27)^, greater incorporation of cooking skills into the DPP could be beneficial for programme engagement and sustained behaviour change^(15,16,28)^. A recent evaluation of Cooking Matters for Diabetes, a cooking intervention for diabetes management, found high acceptability of the intervention and improvements in self-efficacy, diabetes self-management activities and health outcomes^(24,29)^.

We found that DPP participants developed new cooking knowledge and strategies that informed modification of their pre-DPP cooking practices. This suggests co-development of cooking interventions between community members and practitioners can leverage existing community-based knowledge and practices and create an opportunity to design an intervention that is tailored to local needs. Given that our findings were from a self-selected sample of participants, some of whom had participated in cooking classes as part of the DPP, it is possible that the embedded knowledge of cooking skills and techniques may differ in a broader sample of DPP participants. Future research should assess the effect of adding FACS to what is already being learned in the DPP. It will also be important that those developing hands-on cooking skills interventions assess knowledge and practices of participants, using a model such as Cook-Ed^TM(30)^, before developing an intervention.

Findings demonstrate that cost concerns influence food procurement practices. This is consistent with existing research in which people identified cost as a major barrier to aligning their dietary practices with type 2 diabetes prevention and management recommendations^(31)^. Thus, FACS classes should incorporate programming about economical ways to grocery shop and prepare meals as part of prediabetes and diabetes management. Further, though the DPP already encourages participants to shop around the store’s perimeter and read nutritional labels, the curriculum could also focus on ingredients to minimise meal preparation time and strategies for affordable grocery shopping for efficient, healthy meals.

### Limitations

All participants in this study identified as Black women living in Baltimore City or Baltimore County, which may limit generalisability to other racial and ethnic groups, socio-economic groups, and more rural areas. Relatedly, participants in this study were self-selected and may have been interested in helping shape a hands-on cooking skills intervention. The goal of qualitative research is not generalisability, but rather to understand a practice or phenomenon based on the lived experience of those with firsthand knowledge or exposure to the topic, so findings are still applicable to DPP providers and other community-based FACS interventions. Further, self-reported benefits of the DPP and positive changes to cooking-related behaviours could have been influenced by social desirability bias.

## Conclusions

This qualitative study described FACS in a sample of former DPP participants in Baltimore City, MD. Though not explicitly the focus of the DPP, participants changed their cooking practices from pre- to post-DPP participation and adopted several key strategies to facilitate healthier eating. Incorporating a greater focus on FACS in the DPP may be beneficial for helping participants achieve sustained behaviour change to manage prediabetes and prevent development of type 2 diabetes.

## References

[1] Centers for Disease Control and Prevention (2022) The Facts, Stats, and Impacts of Diabetes. https://www.cdc.gov/diabetes/library/spotlights/diabetes-facts-stats.html (accessed April 2022).

[2] Gaskin DJ , Thorpe RJ Jr , McGinty EE et al. (2014) Disparities in diabetes: the nexus of race, poverty, and place. Am J Public Health 104, 2147–2155. 24228660 10.2105/AJPH.2013.301420 PMC4021012

[3] Diabetes Prevention Program Research G (2002) The Diabetes Prevention Program (DPP): description of lifestyle intervention. Diabetes Care 25, 2165–2171. 12453955 10.2337/diacare.25.12.2165 PMC1282458

[4] American Diabetes Association Professional Practice C (2022) 3. Prevention or delay of type 2 diabetes and associated comorbidities: standards of medical care in diabetes-2022. Diabetes Care 45, S39–S45. 34964876 10.2337/dc22-S003

[5] Centers for Disease Control and Prevention (2021) Type 2 Diabetes. https://www.cdc.gov/diabetes/basics/type2.html#:∼:text=More%20than%2037%20million%20Americans,them%20have%20type%202%20diabetes (accessed April 2022).

[6] Wolfson JA , Lahne J , Raj M et al. (2020) Food agency in the United States: associations with cooking behavior and dietary intake. Nutrients 12, 877. 32213985 10.3390/nu12030877 PMC7146410

[7] Lavelle F , McGowan L , Hollywood L et al. (2017) The development and validation of measures to assess cooking skills and food skills. Int J Behav Nutr Phys Act 14, 118. 28865452 10.1186/s12966-017-0575-y PMC5581465

[8] Centers for Disease Control and Prevention (2020) Curricula and Handouts. https://www.cdc.gov/diabetes/prevention/resources/curriculum.html (accessed April 2022).

[9] Lavelle F , McGowan L , Spence M et al. (2016) Barriers and facilitators to cooking from ‘scratch’ using basic or raw ingredients: a qualitative interview study. Appetite 107, 383–391. 27567551 10.1016/j.appet.2016.08.115

[10] McGowan L , Pot GK , Stephen AM et al. (2016) The influence of socio-demographic, psychological and knowledge-related variables alongside perceived cooking and food skills abilities in the prediction of diet quality in adults: a nationally representative cross-sectional study. Int J Behav Nutr Phys Act 13, 111. 27782841 10.1186/s12966-016-0440-4 PMC5080680

[11] Wolfson JA , Leung CW & Richardson CR (2020) More frequent cooking at home is associated with higher Healthy Eating Index-2015 score. Public Health Nutr 23, 2384–2394. 31918785 10.1017/S1368980019003549 PMC11374573

[12] Mills S , Brown H , Wrieden W et al. (2017) Frequency of eating home cooked meals and potential benefits for diet and health: cross-sectional analysis of a population-based cohort study. Int J Behav Nutr Phys Act 14, 109. 28818089 10.1186/s12966-017-0567-y PMC5561571

[13] Garcia AL , Reardon R , McDonald M et al. (2016) Community interventions to improve cooking skills and their effects on confidence and eating behaviour. Curr Nutr Rep 5, 315–322. 27882266 10.1007/s13668-016-0185-3 PMC5097072

[14] Reicks M , Kocher M & Reeder J (2018) Impact of cooking and home food preparation interventions among adults: a systematic review (2011–2016). J Nutr Educ Behav 50, 148–172. 28958671 10.1016/j.jneb.2017.08.004

[15] Polak R , Tirosh A , Livingston B et al. (2018) Preventing type 2 diabetes with home cooking: current evidence and future potential. Curr Diab Rep 18, 99. 30218282 10.1007/s11892-018-1061-x

[16] Archuleta M , Vanleeuwen D , Halderson K et al. (2012) Cooking schools improve nutrient intake patterns of people with type 2 diabetes. J Nutr Educ Behav 44, 319–325. 22572403 10.1016/j.jneb.2011.10.006

[17] Harper D (2002) Talking about pictures: a case for photo elicitation. Visual Stud 17, 13–26.

[18] Mills S , White M , Wrieden W et al. (2017) Home food preparation practices, experiences and perceptions: a qualitative interview study with photo-elicitation. PLoS One 12, e0182842. 28854196 10.1371/journal.pone.0182842 PMC5576640

[19] Tong A , Sainsbury P & Craig J (2007) Consolidated criteria for reporting qualitative research (COREQ): a 32-item checklist for interviews and focus groups. Int J Qual Health Care 19, 349–357. 17872937 10.1093/intqhc/mzm042

[20] Wolfson JA , Bleich SN , Smith KC et al. (2016) What does cooking mean to you?: perceptions of cooking and factors related to cooking behavior. Appetite 97, 146–154. 26654888 10.1016/j.appet.2015.11.030

[21] Charmaz K (2006) Constructing Grounded Theory: A Practical Guide Through Qualitative Analysis. Thousand Oaks, CA: SAGE Publications Inc.

[22] Birks M , Chapman Y & Francis K (2008) Memoing in qualitative research: probing data and processes. J Res Nursing 13, 68–75. 10.1177/17449871251386323 PMC12602296 41229605

[23] Vidgen HA & Gallegos D (2014) Defining food literacy and its components. Appetite 76, 50–59. 24462490 10.1016/j.appet.2014.01.010

[24] Williams A , Shrodes JC , Radabaugh JN et al. (2022) Outcomes of cooking matters for diabetes: a 6-week randomized, controlled cooking and diabetes self-management education intervention. J Acad Nutr Diet 123, 477–491. 10.1016/j.jand.2022.07.021 PMC10862535 35961614

[25] McGowan L , Caraher M , Raats M et al. (2017) Domestic cooking and food skills: a review. Crit Rev Food Sci Nutr 57, 2412–2431. 26618407 10.1080/10408398.2015.1072495

[26] Srebnik D , Chwastiak LA , Russo J et al. (2015) A pilot study of the diabetes prevention program on weight loss for adults at community mental health centers. Psychiatr Serv 66, 200–203. 25642616 10.1176/appi.ps.201300576

[27] Realmuto L , Kamler A , Weiss L et al. (2018) Power up for health-participants’ perspectives on an adaptation of the national diabetes prevention program to engage men. Am J Mens Health 12, 981–988. 29540130 10.1177/1557988318758786 PMC6131458

[28] Zong G , Eisenberg DM , Hu FB et al. (2016) Consumption of meals prepared at home and risk of type 2 diabetes: an analysis of two prospective cohort studies. PLoS Med 13, e1002052. 27379673 10.1371/journal.pmed.1002052 PMC4933392

[29] Shrodes JC , Williams A , Nolan TS et al. (2022) Feasibility of cooking matters for diabetes: a 6-week randomized, controlled cooking and diabetes self-management education intervention. J Acad Nutr Diet 123, 492–503. 10.1016/j.jand.2022.07.020 PMC10909744 35944873

[30] Asher RC Jakstas T , Wolfson JA et al. (2020) Cook-Ed(TM): a model for planning, implementing and evaluating cooking programs to improve diet and health. Nutrients 12, 2011. 32640756 10.3390/nu12072011 PMC7400850

[31] Byrne C , Kurmas N , Burant CJ et al. (2017) Cooking classes: a diabetes self-management support intervention enhancing clinical values. Diabetes Educ 43, 600–607. 29047323 10.1177/0145721717737741

[32] United States Census Bureau (2022) Quick Facts – Baltimore City, Maryland; Baltimore County, Maryland. https://www.census.gov/quickfacts/fact/table/baltimorecitymaryland,baltimorecountymaryland/PST045222 (accessed April 2023).

